# Development of a Peptide-Based Photoimmunotherapy Drug Targeting PD-L1

**DOI:** 10.3390/molecules31020302

**Published:** 2026-01-14

**Authors:** Takuya Otani, Naoya Kondo, Ayaka Kanai, Hirofumi Hanaoka

**Affiliations:** Near InfraRed Photo-ImmunoTherapy Research Institute, Kansai Medical University, Hirakata 573-1010, Osaka, Japan; ootani.tky@kmu.ac.jp (T.O.); kondo.ny@kmu.ac.jp (N.K.); kanaia@hirakata.kmu.ac.jp (A.K.)

**Keywords:** near-infrared photoimmunotherapy, PD-L1, WL12, cyclic peptide, anticancer drug

## Abstract

Near-infrared photoimmunotherapy (NIR-PIT) has recently attracted attention as a highly selective cancer treatment, with good treatment outcomes observed from the only antibody-based drug currently available for clinical use. However, since only a single agent is currently used clinically and the development of new antibodies is costly, exploring other therapeutic modalities is important. In this study, we investigated a novel peptide-based PIT drug targeting programmed death-ligand 1 (PD-L1), which is overexpressed in many types of cancer. The WL12 peptide, which is known to bind to PD-L1, was conjugated with the photoabsorber IRDye700DX (IR700), and its usefulness was evaluated in vitro and in vivo. In therapeutic experiments on PD-L1-positive cells, NIR-PIT with WL12-IR700 induced PIT-like morphological changes in cells and reduced cancer cell viability in an NIR light dose- and drug concentration-dependent manner. In vivo experiments showed significant suppression of tumor growth and an extended overall survival rate. These results indicate that the developed peptide-based drug can be used for PD-L1-targeted NIR-PIT.

## 1. Introduction

Near-infrared photoimmunotherapy (NIR-PIT) is a new targeted treatment for cancer [1,2,3,4]. With this strategy, the conjugate of target-directed molecules that bind to cancer cells and the photoabsorber IRDye700DX (IR700) is used as the drug. By irradiating NIR light after the drug binds to the target molecule on cancer cells, these cells are selectively killed. NIR-PIT involves the administration of a PIT drug, binding the drug to its target on cancer cells, and subsequent irradiation with NIR light. The target-directed molecules used in NIR-PIT are primarily monoclonal antibodies that selectively bind to specific antigens expressed on the surface of cancer cells. Irradiation with NIR light at approximately 690 nm from the outside of the body induces a structural change in IR700, which imposes stress on membrane proteins, in turn causing cell membrane damage. Consequently, target cells immediately swell, rupture, and undergo necrosis-like cell death [5,6]. Owing to this mechanism, NIR-PIT exhibits high specificity, immediate therapeutic efficacy, and low invasiveness because it relies solely on light, and light can be reapplied according to the therapeutic response. Moreover, the cetuximab-IR700 conjugate targeting epidermal growth factor receptor (EGFR) was approved in Japan in 2020 and has shown promising clinical results [7,8,9]. However, this is the only antibody-based drug currently in clinical use, with the development of new antibody-based drugs for novel targets being costly and time-consuming.

Programmed death-ligand 1 (PD-L1) is an important molecule involved in immune suppression; it has attracted attention as a target for cancer immunotherapy. As an immune checkpoint molecule, PD-L1 is a cell membrane protein belonging to the B7 family and is mainly expressed in cancer and antigen-presenting cells [10,11,12]. It inhibits T cell activation by binding to PD-1 receptors on the surface of T cells. It inhibits T cell proliferation, cytokine production, and cytotoxic activity, thereby suppressing autoimmune responses and enabling cancer cells to evade the immune system. PD-L1 is overexpressed in many types of cancer, including non-small cell lung cancer (NSCLC), gastric cancer, and breast cancer [13,14]. Multiple immune checkpoint inhibitors targeting the PD-1/PD-L1 axis have been approved as cancer treatments [14,15,16]. In addition, various treatments targeting PD-L1 are being developed [17,18,19]. Moreover, NIR-PIT with anti-PD-L1 antibodies has demonstrated sufficient therapeutic effects in mouse xenograft models [20,21]. Therefore, the clinical application of NIR-PIT for targeting PD-L1 is highly desirable. However, antibody-based drugs are costly and their development is challenging. In contrast, peptides can be chemically synthesized at low production costs [22]. Additionally, because of their small size, they exhibit high permeability within tumors, enable more uniform and deeper penetration than antibodies, and have the potential to enhance the therapeutic effects of NIR-PIT [23]; their efficacy in photoimmunotherapy has been reported [24].

**Figure 1:**
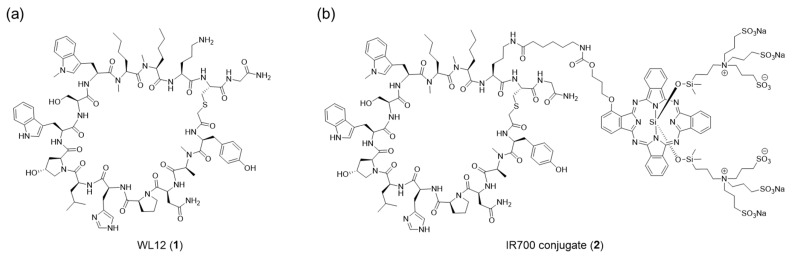
Structures of (**a**) WL12 and (**b**) an IR700 conjugate.

WL12 is a synthetic cyclic peptide with high affinity for PD-L1 (Figure 1a) [25]. It has a molecular mass of approximately 2 kDa and has recently been investigated as a positron emission tomography (PET) imaging probe following labeling with ^64^Cu or ^68^Ga [25,26,27]. They have been reported to have high accumulation in cancer cells and favorable pharmacokinetics. Furthermore, ^68^Ga-NOTA-WL12 has been used in a first-in-human study in patients with NSCLC, enabling clear and safe visualization of cancers [28]. As WL12 is a cyclic peptide containing *N*-methylation, it is expected to have high metabolic stability, affinity, and selectivity for its targets as previously reported [29,30,31]. These properties mitigate typical peptide liabilities such as rapid degradation and lower affinity and selectivity than antibodies. Thus, WL12 is a promising target-directed molecule for NIR-PIT. In the present study, we developed a novel PIT drug using WL12 as a PD-L1 targeting scaffold (Figure 1b). We synthesized a WL12-IR700 conjugate and evaluated its performance as an NIR-PIT drug in vitro and in vivo.

## 2. Results and Discussion

### 2.1. Synthesis of the WL12-IR700 Conjugate

**Scheme 1:**
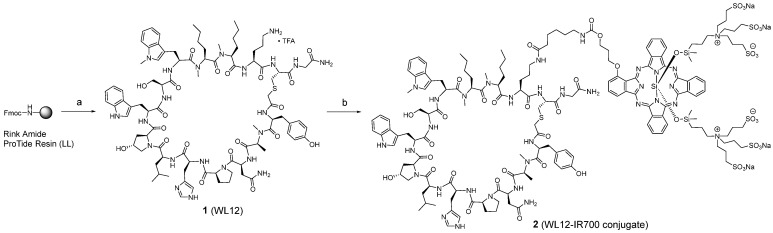
Synthesis of the WL12-IR700 conjugate (**2**). Reagents and conditions: (**a**) (i) Fmoc-SPPS; (ii) *N*-(chloroacetoxy)succinimide, DMF, 20–25 °C, 40 min; (iii) TFA, EDT, TIS, H_2_O, 20–25 °C, 2 h; (iv) TEA, MeCN, H_2_O, 40 °C, 2 h (18% from the resin); (**b**) IR700 NHS ester, DIPEA, DMSO, 40 °C, 19 h (67% using IR700 NHS ester as the starting material).

Synthesis of the WL12-IR700 conjugate (**2**) is shown in Scheme 1. WL12 (**1**) was obtained via 9-fluorenylmethyloxycarbonyl (Fmoc) solid-phase peptide synthesis (SPPS) using a microwave peptide synthesizer, chloroacetylation at the N-terminus, deprotection, cleavage from the resin, and an intramolecular cyclization reaction. The amino group of the ornithine residue in **1** was then reacted with IR700 *N*-hydroxysuccinimide ester (IR700 NHS ester) to obtain WL12-IR700. WL12 possesses only one amine group that can react with NHS ester. The WL12 peptide employed in this study has been extensively characterized in previous nuclear medicine studies, with its high affinity and selectivity for PD-L1 established [25,26]. Docking studies further indicated that the amino group of the ornithine residue on WL12, which is used for chelator attachment in radiolabeling, points away from the PD-L1 binding interface [25,26]. Similarly, IR700 was introduced into the ornithine residue. Analysis confirmed that the expected 1:1 conjugated compound of WL12 and IR700 was obtained (Figure S1).

### 2.2. Binding to Cancer Cells

**Figure 2:**
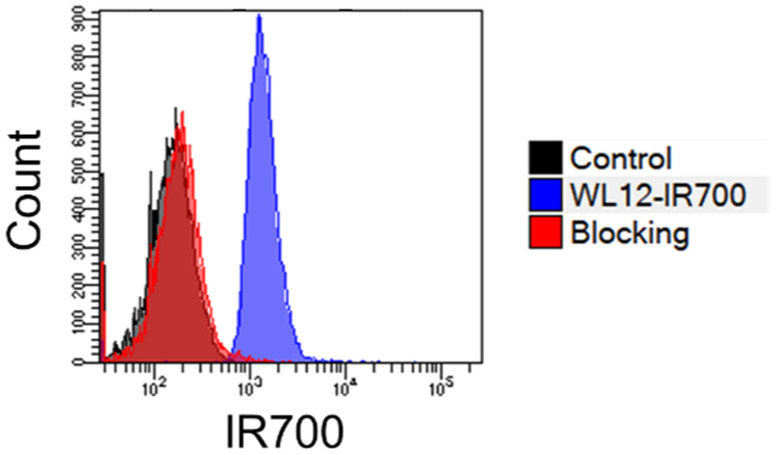
Flow cytometry analysis of WL12-IR700 following binding to MDA-MB-231 cells. Control represents cells treated with PBS without WL12-IR700 (black), WL12-IR700 represents cells treated with a solution of WL12-IR700 (blue), and Blocking represents cells pretreated with WL12 followed by treatment with a solution of WL12-IR700 (red).

To evaluate the binding of WL12-IR700 to PD-L1 on cancer cell membranes, the fluorescence of WL12-IR700 bound to the cells was measured using a flow cytometer (Figure 2); WL12-IR700 bound to MDA-MB-231 cells, which highly express PD-L1 [32]. Since flow cytometry was performed after incubation for 10 min at 4 °C, drug internalization did not occur, indicating binding to the cell surface. Although the bulky IR700 conjugate may have reduced WL12 affinity for its target, it demonstrated sufficient binding to target-positive cells. In contrast to antibodies in which IR700 is randomly introduced at multiple lysine residues, the WL12 scaffold permits site-selective attachment of IR700 at the distal amine, which is expected to preserve binding while conferring light-activatable cytotoxicity. Additionally, whether WL12-IR700 specifically binds to MDA-MB-231 cells was evaluated. When a competitive inhibition experiment was performed using an excess amount of WL12, as a blocking agent, it exhibited fluorescence comparable to the control.

Furthermore, in the competitive inhibition experiment of pretreatment with WL12, the binding of WL12-IR700 was almost completely inhibited. This suggests that WL12-IR700 specifically binds to MDA-MB-231 cells. Similar specificity has been reported for other WL12 derivatives [25,26].

### 2.3. Microscopic Observation

**Figure 3:**
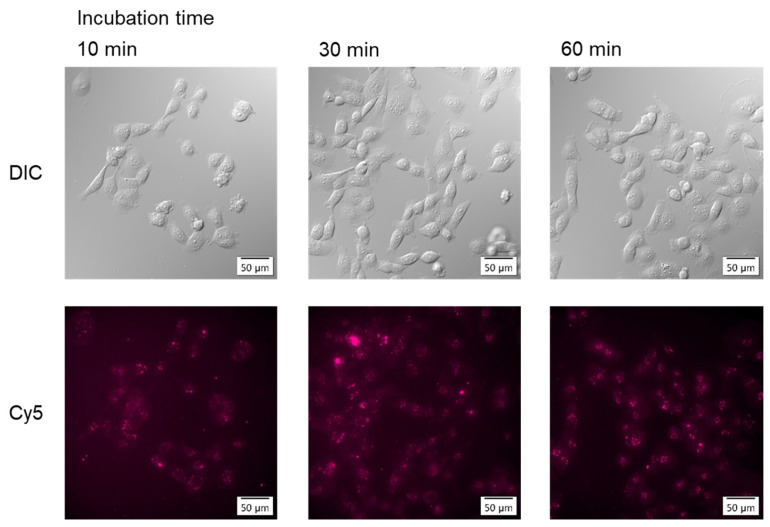
Microscopic observations of cells with WL12-IR700. The upper panels are DIC images, and the lower panels are fluorescence images.

Binding of WL12-IR700 to MDA-MB-231 cells and morphological changes in cells before and after NIR light irradiation were observed using a fluorescence microscope. In Figure 3, the upper panels show differential interference contrast (DIC) images, and the lower panels show fluorescence images of IR700 fluorescence. The drug was initially distributed throughout the entire cell; however, after 60 min, a spot-like localized distribution in the cell was observed. This suggests that it internalized over time, leading to increased fluorescence in the lysosomal fraction.

**Figure 4:**
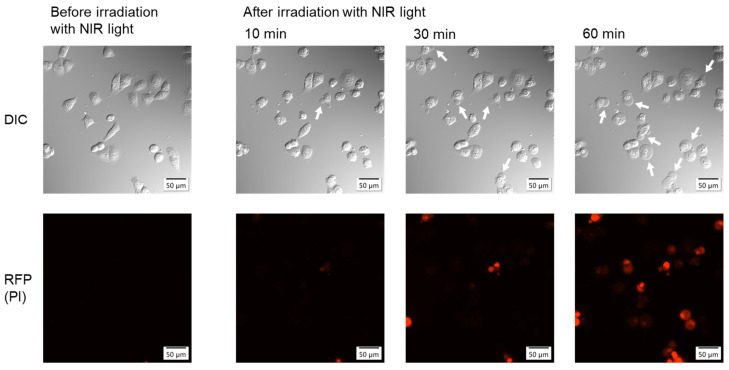
Microscopic observations of cells before and after NIR light irradiation with WL12-IR700. The upper panels are DIC images, and the lower panels are fluorescence images. The white arrows indicate bleb formation.

In Figure 4, the upper panels show DIC images. Morphological changes, such as bleb formation and swelling, were observed in some cells 10 min after irradiation and in most cells 60 min after irradiation. Bleb formation and swelling are characteristic morphological changes observed in NIR-PIT with antibody-based drugs [1,2,3]. These changes arise from IR700 structural changes on the cell surface upon NIR irradiation, in turn causing membrane damage [3,4,5,6]. Consistent with this mechanism, WL12-IR700 elicited rapid bleb formation and cell swelling after irradiation. In the lower panels, the fluorescence of propidium iodide (PI), which intercalates into nuclear DNA, was observed in the cells, indicating cell membrane destruction. The number of cells positive for red fluorescence also increased over time after irradiation, similar to the morphological changes. Similarly, cell death based on this mechanism has been observed using a peptide-based drug targeting EGFR [24], suggesting that peptides have significant potential as scaffolds for NIR-PIT. Although no evidence currently exists, considering the size of the peptide-based drug, it is speculated that this significant membrane damage may have been a consequence of aggregation involving not only the drug, but also the target membrane protein.

### 2.4. Cell Viability Assays

**Figure 5:**
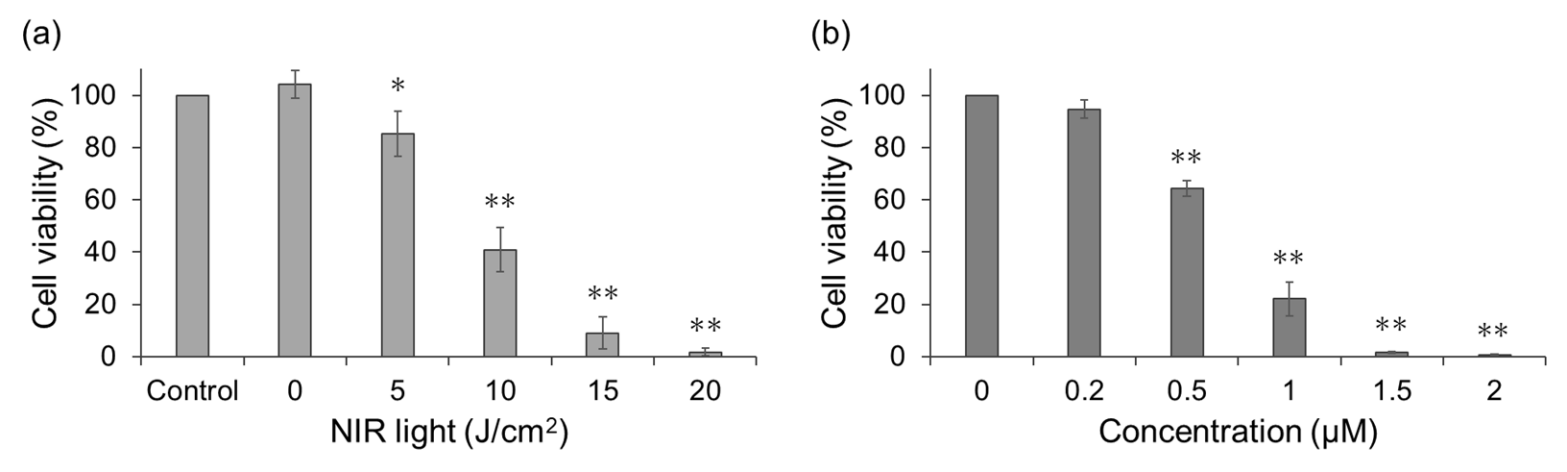
Cell viability assessed by CCK-8 assays. (**a**) NIR light dose-dependent cell viability after treatment with WL12-IR700 (concentration: 2 μM). Control represents the untreated group, and its cell viability was deemed as 100%. * *p* < 0.05, ** *p* < 0.01 with respect to treatment with 0 J/cm^2^ (Welch’s *t*-test). (**b**) Drug concentration-dependent cell viability after treatment with WL12-IR700 (NIR light: 20 J/cm^2^). Cell viability of 0 μM was deemed as 100%. ** *p* < 0.01 with respect to treatment with 0 μM (Welch’s *t*-test).

To quantify cytotoxicity, cell viability was assessed using the Cell Counting Kit-8 (CCK-8) assay (Figure 5). First, the NIR light dose-dependent cell viability following treatment with WL12-IR700 was examined (Figure 5a). Cell viability decreased significantly compared with that of the non-irradiated control, and this decrease was dependent on the dose of NIR light. Next, drug concentration-dependent cell viability was examined (Figure 5b). Irradiation experiments were performed at various drug concentrations (0.2–2 µM), and cell viability decreased in a drug-concentration-dependent manner.

The cytotoxicity of NIR-PIT scales with the surface density of IR700 on the membrane and the delivered light dose; light dose–response behavior has been replicated across multiple antigens and conjugate classes [1,2,20,21]. We previously revealed the light dose-dependence of a peptide-based PIT drug targeting EGFR [24]. Likewise, this study showed NIR light dose- and drug concentration-dependent cell death in NIR-PIT with WL12-IR700. These findings support the notion that WL12-IR700 operates via a canonical NIR-PIT mechanism.

### 2.5. In Vivo Accumulation Study

**Figure 6:**
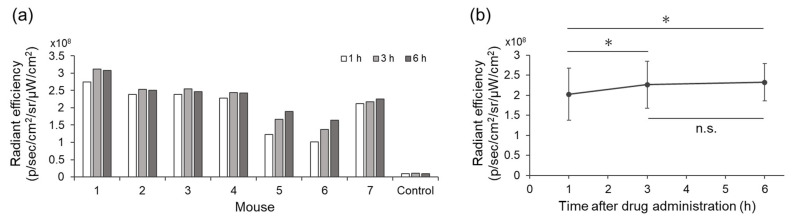
Fluorescence intensity in the tumor after injection of WL12-IR700. (**a**) Radiant efficiency of individual mice (Mouse 1–7) and an uninjected mouse (Control). (**b**) Average radiant efficiency of drug-administered mice. * *p* < 0.05, n.s.: not significant (paired *t*-test adjusted by Holm method after repeated-measures ANOVA).

To evaluate the usefulness of WL12-IR700 in vivo, mice bearing MDA-MB-231 tumors were established. IR700 fluorescence in the tumor was quantified using IVIS after the intravenous injection of WL12-IR700 to determine the optimal timing of NIR light irradiation in therapeutic experiments (Figure 6 and Figure S3). Radiant efficiency in the tumor at 3 and 6 h post-injection showed significantly higher fluorescence intensity than that at 1 h (Figure 6b). A strong IR700 signal was also detected in the kidneys (Figure S3), a distribution pattern consistent with low-molecular weight ligands that undergo rapid renal clearance [33,34]. In NIR-PIT, only the areas irradiated with light showed cell damage, suggesting that accumulation in the kidneys is not a major problem. To this end, tumor accumulation level was the most important factor, and the results showed a significantly higher accumulation at the tumor site at 3 and 6 h compared to 1 h post-administration, whereas no significant difference was observed between 3 and 6 h. In addition, given that NIR-PIT exerts its therapeutic effect when the drug is present on the cell membrane surface [35] and considering that PD-L1-bound peptides undergo internalization over time as shown Figure 3, we determined that 3 h post-administration was the optimal time for NIR light irradiation.

Additionally, only IR700 dye, lacking the WL12 ligand, was administered to tumor-bearing mice (Figure S3), and did not accumulate in the tumor. It was revealed that WL12 functions as a target-directed molecule even in an animal model, implying that peptides can deliver IR700 to target sites even in vivo.

### 2.6. In Vivo Therapeutic Experiment

**Figure 7:**
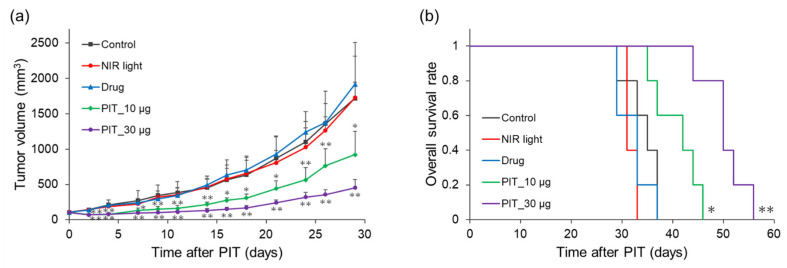
Therapeutic effect of NIR-PIT with WL12-IR700 in tumor-bearing mice. (**a**) Average of tumor volume in each group. * *p* < 0.05, ** *p* < 0.01 with respect to the control group (Dunnett’s multiple comparison test after one-way ANOVA). (**b**) Overall survival rate for each group. The mice with a tumor volume > 2000 mm^3^ were treated as dead. * *p* < 0.05, ** *p* < 0.01 with respect to the control group (log-rank test).

Therapeutic effects were evaluated in mice bearing MDA-MB-231 tumors (Figure 7). The tumor volumes in the NIR light and drug groups increased similarly to those in the control group. In contrast, the two PIT groups significantly suppressed the increase in tumor volume compared to that in the control group from early time points after treatment initiation (Figure 7a). Overall survival was not prolonged in the NIR light and drug groups, whereas both PIT groups showed significant survival extension (Figure 7b). The median survival times in control, NIR light, drug, PIT_10 µg, and PIT_30 µg groups were 35, 31, 33, 42, and 50 days, respectively. A certain degree of difference in the therapeutic effect was observed between the different doses. A superior therapeutic effect was observed with dose escalation, which was consistent with the in vitro results. Further optimization of the drug quantity and irradiation timing could enhance the fraction of IR700 residing on the cell membrane during treatment, thereby improving therapeutic outcomes.

Dose selection for in vivo experiments was based on the IR700-equivalent conversion of a clinically established antibody-based drug [2]. The reference antibody-based drug is dosed at 100 µg with three IR700 dyes per antibody. When normalized by IR700 equivalents, the WL12-IR700 developed herein corresponds to approximately 10 µg. Therapeutic experiments were performed using 10 µg and escalated doses of 30 µg (Figure 7). Both PIT groups showed significantly suppressed tumor volume increases compared to the control group from the early time points after NIR-PIT. Although the cancer cell lines were different, similar results were obtained with the antibody-based NIR-PIT targeting PD-L1 [20,21]. Thus, peptide-based NIR-PIT is likely to have a similar effect to that utilizing antibody. Peptide-based drugs are less expensive than their antibody-based counterparts, making it possible to increase their dosage. In addition, a PET study of WL12 in ongoing clinical trials is likely to be useful with this optimization [28].

From a translational perspective, peptide-based NIR-PIT offers several distinct practical advantages over antibody-based drugs, including synthetic accessibility, reduced production costs, batch-to-batch homogeneity through site-selective labeling, rapid tumor accumulation kinetics, better tumor penetration, and accelerated systemic clearance that may mitigate photosensitivity risks while facilitating flexible dosing schedules or repeated administrations [22,36,37,38]. Collectively, these results position WL12-IR700 as a promising PD-L1-targeted PIT candidate and motivate systematic studies to optimize its pharmacokinetics, membrane residency at irradiation, and treatment parameters to fully leverage peptide-driven NIR-PIT in clinical practice.

This study has some limitations. First, only one cell line was used during these investigations. However, since the cytotoxic mechanism of NIR-PIT involves membrane damage after NIR light irradiation, the therapeutic effects of NIR-PIT are thought to occur independently of cell type. The therapeutic effects of NIR-PIT on other cell lines have been demonstrated using antibody-based drugs targeting PD-L1, and therapeutic effects on various cell lines have been revealed with NIR-PIT targeting other molecules [1,2,20]. Second, nude mice lacking T cells were used. Because PD-L1 binds to PD-1 on T cells, the presence or absence of T cells may have affected the results. However, NIR-PIT with antibody-based drugs targeting PD-L1 has demonstrated sufficient therapeutic effects, even in an immunocompetent syngeneic model [21]; therefore, WL12-IR700 would demonstrate therapeutic efficacy even in the presence of T cells. We plan to use an immunocompetent syngeneic model to investigate immune responses, including immunogenic cell death markers, T cell activation, and abscopal effects. Third, the therapeutic effect of WL12-IR700 was not directly compared with that of the antibody-based drug in this study. However, since treatment efficacy varies depending on several factors such as the administered drug dose and timing of light irradiation within the treatment protocol, it is difficult to evaluate superiority at this stage. This study aimed only to demonstrate that NIR-PIT is fully feasible using a peptide-based drug, leaving comparison with antibodies as a future task.

## 3. Materials and Methods

### 3.1. Synthesis and Characterization of the WL12-IR700 Conjugate

#### 3.1.1. General

Amino acid derivatives were purchased from Watanabe Chemical Inc., Ltd. (Hiroshima, Japan). IR700 NHS ester was purchased from EOS MedChem (Jinan City, China). Other reagents were purchased from FUJIFILM Wako Pure Chemical Corporation (Osaka, Japan), Tokyo Chemical Industry Co., Ltd. (Tokyo, Japan), and CEM Corporation (Matthews, NC, USA) and were used without further purification. Preparative high performance liquid chromatography (HPLC) was performed using a COSMOSIL 5C18-ARII column (20 ID × 250 mm, Nacalai Tesque, Inc.; Kyoto, Japan) with a linear gradient of MeCN in 0.1% aqueous trifluoroacetic acid (TFA) or MeCN in 0.1 M aqueous triethylammonium acetate (TEAA) at a flow rate of 9.0 mL/min on an LC-20AR system (OD, 220 or 680 nm, Shimadzu Corporation; Kyoto, Japan). For analytical HPLC, a COSMOSIL 5C18-ARII column (4.6 ID × 150 mm, Nacalai Tesque, Inc.) was employed with a linear gradient of MeCN in 0.1% aqueous TFA or MeCN in 0.1 M aqueous TEAA at a flow rate of 0.9 mL/min on an LC-20AD system (OD, 220 or 680 nm, Shimadzu Corporation). Electrospray ionization (ESI) mass spectra (MS) were recorded using an LCMS-8050 instrument (Shimadzu Corporation). The absorption and fluorescence spectra were recorded using a V-730BIO spectrophotometer (JASCO, Tokyo, Japan) and an FP-8650 spectrofluorometer (JASCO), respectively.

#### 3.1.2. Fmoc SPPS

Fmoc SPPS was performed using a Liberty Blue automated microwave peptide synthesizer (CEM Corporation). The amino acids were coupled in 5-fold excess using 0.25 M solution of *N*, *N*’-diisopropylcarbodiimide in *N*, *N*-dimethylformamide (DMF) and 0.25 M solution of Oxyma Pure in DMF. Single coupling was performed for 2 min at 90 °C, and double coupling was performed twice using a single coupling protocol. Fmoc deprotections were performed for 1 min at 90 °C using a 20% (*v*/*v*) solution of piperidine in DMF. Between all the steps, the resin was washed four times with DMF.

#### 3.1.3. Synthesis of Peptide 1 (WL12)

The peptidyl resin was synthesized with Rink Amide ProTide Resin (LL) (0.025 mmol scale) by Fmoc SPPS using a microwave peptide synthesizer. All reactions after the second methylnorleucine reaction were performed by double coupling. After deprotection of the Fmoc group of the N-terminal tyrosine, the peptidyl resin was added to a 0.5 M solution of *N*-(chloroacetoxy)succinimide in DMF (1.0 mL) and stirred for 40 min at 20–25 °C. The resin was washed with DMF, MeOH, CHCl_3_, and Et_2_O. To cleave the resin and deprotect, the peptidyl resin was treated with TFA/1,2-ethanedithiol (EDT)/triisopropylsilane (TIS)/H_2_O [2.0 mL, 92.5:2.5:2.5:2.5 (*v*/*v*/*v*/*v*)] for 2 h at 20–25 °C. After filtering and concentrating under reduced pressure, the residue was poured into ice-cold Et_2_O. The resulting powder was collected by centrifugation and washed three times with ice-cold Et_2_O. The crude product was dissolved in 50% MeCN/H_2_O (10 mL), triethylamine (TEA; 100 µL) was added, and the mixture was stirred for 2 h at 40 °C. After concentrating MeCN under reduced pressure, the residue was lyophilized. The crude product was purified using preparative reverse phase (RP)-HPLC to obtain peptide **1** (9.02 mg, 18% from the resin) as a freeze-dried white powder. The ESI-MS *m*/*z* calculated for C_91_H_128_N_22_O_20_S: 1880.9 (TFA desalted) recorded a peak at [M+2H]^2+^ *m*/*z*: 941.9.

#### 3.1.4. Synthesis of Conjugate 2 (WL12-IR700)

Peptide **1** (1.80 mg, 0.901 µmol) and *N*, *N*-diisopropylethylamine (DIPEA; 0.078 µL, 0.450 µmol) were added to a solution of IR700 NHS ester (0.88 mg, 0.450 µmol) in dimethyl sulfoxide (DMSO; 100 µL) and stirred for 19 h at 40 °C. The mixture was diluted with 40% MeCN/H_2_O. The crude product was purified by preparative RP-HPLC and passed through an ion exchange resin (Amberlite IR120B Na, ORGANO CORPORATION; Tokyo, Japan) to obtain conjugate **2** (1.12 mg, 67%, using IR700 NHS ester as the starting material) as a freeze-dried blue powder. The ESI-MS *m*/*z* calculated for C_161_H_219_N_33_O_44_S_7_Si_3_^4–^: 3626.3 (Na^+^ desalted) recorded a peak at [M]^4–^ *m*/*z*: 907.3 and [M+H]^3–^ *m*/*z*: 1209.9. The absorption and fluorescence spectra are shown in Figure S2.

### 3.2. Cell Culture

MDA-MB-231 cells were obtained from American Type Culture Collection (ATCC; Manassas, VA, USA). The cells were cultured in Dulbecco’s modified Eagle’s medium (DMEM, FUJIFILM Wako Pure Chemical Corporation) supplemented with 10% (*v*/*v*) fetal bovine serum (FBS, Biowest; Nuaillé, France) and 2% (*v*/*v*) penicillin-streptomycin solution (FUJIFILM Wako Pure Chemical Corporation) and incubated at 37 °C with 95% air and 5% CO_2_.

### 3.3. Flow Cytometry

Approximately 1 × 10^6^ MDA-MB-231 cells were suspended in Roswell Park Memorial Institute (RPMI) medium (FUJIFILM Wako Pure Chemical Corporation) supplemented with 10% (*v*/*v*) FBS (control, 100 µL) or a 5 µM solution of WL12-IR700 in RPMI medium supplemented with 10% (*v*/*v*) FBS (100 µL). After incubation for 10 min at 4 °C, the samples were centrifuged, the supernatant was removed, and 1 mL of phosphate-buffered saline (PBS, FUJIFILM Wako Pure Chemical Corporation) was added. The blocking sample was treated with a 250 µM solution of WL12 for 30 min before treatment with WL12-IR700, and the same procedure as above was performed. The samples were filtered through a 35 µm strainer cap (MTC Bio; Sayreville, NJ, USA) and then analyzed using a BD FACSymphony A1 flow cytometer (BD Biosciences; Franklin Lakes, NJ, USA).

### 3.4. Microscopic Observation

Approximately 2 × 10^5^ MDA-MB-231 cells in DMEM were seeded in a glass-bottom dish and incubated overnight at 37 °C in a 5% CO_2_ atmosphere. To observe binding to cells, after removal of the medium and washing with RPMI medium supplemented with 10% (*v*/*v*) FBS, the cells were incubated with a 5 µM solution of WL12-IR700 in RPMI medium with 10% (*v*/*v*) FBS (1 mL) at 37 °C. After removal of the medium and washing with RPMI medium supplemented with 10% (*v*/*v*) FBS, the cells were observed using an IX83 microscope (Olympus; Tokyo, Japan). To observe morphological changes, after removal of the medium and washing with RPMI medium, the cells were incubated with a 5 µM solution of WL12-IR700 in RPMI medium (1 mL) for 10 min at 37 °C. The solution was then replaced with RPMI medium, and 1 mg/mL solution of PI (FUJIFILM Wako Pure Chemical Corporation) in PBS was added. The cells were then irradiated with NIR light (20 J/cm^2^) through a 690 nm excitation filter (Semrock; Rochester, NY, USA) attached to an IX83 microscope.

### 3.5. Cell Viability Assays

Approximately 2 × 10^4^ MDA-MB-231 cells in DMEM were seeded in 24-well plates and incubated overnight at 37 °C in a 5% CO_2_ atmosphere. After removal of the medium and washing with RPMI medium, the cells were incubated with a solution of WL12-IR700 in RPMI medium (500 µL) for 10 min at 37 °C. The cells were then irradiated with NIR light using an M10-QD303 2CH regulated DC power supply (MCP Lab Electronics; Gdańsk, Poland), an AD-8724D DC power supply (A&D, Tokyo, Japan), and an LED package (Ushio Inc.; Tokyo, Japan). After removal of the solution and washing with PBS, the cells were incubated with DMEM for 24 h at 37 °C. The medium was then removed and the cells were washed with RPMI medium. CCK-8 solution (DOJINDO LABORATORIES, Kumamoto, Japan) in RPMI medium was added to each well and the cells were incubated. After incubation for 3 h at 37 °C, 100 µL of the solution was transferred to a 96-well plate. Absorbance was measured at 450 nm using a GloMax Discover microplate reader (Promega Corporation; Madison, WI, USA). The experiment was independently performed in triplicate.

### 3.6. Animal and Tumor Xenograft Model

All animal experiments were approved by the Animal Experimentation Committee of Kansai Medical University and performed in accordance with the Rules and Regulations for Animal Experimentation stated by Kansai Medical University. Five-week-old female BALB/c nude mice were purchased from The Jackson Laboratory Japan, Inc. (Kanagawa, Japan) and acclimatized for 10 days. Approximately 3 × 10^6^ MDA-MB-231 cells were suspended in 50 µL of PBS and 50 µL of Matrigel (Corning Life Sciences; Bedford, MA, USA), and injected subcutaneously in the right flank of each mouse. The tumor volumes were calculated using Equation (1).

Tumor volume = Length × Width × Width × 0.5(1)

### 3.7. In Vivo Accumulation Study

When tumor volumes of the eight mice reached 100–200 mm^3^, 30 µg/mouse of WL12-IR700 was administered intravenously to seven mice. The control mouse was not administered WL12-IR700. Imaging using an IVIS Spectrum (PerkinElmer, Waltham, MA, USA) was performed at 1, 3, and 6 h post-administration (Ex: 710 nm, Em: 780 nm, exposure time: 1 s). Five mice administered only IR700 dye were similarly prepared, and were administered an 80 µM solution of IR700 dye (100 µL). The radiant efficiency, defined as the fluorescence emission radiance per incident excitation intensity, of the tumor was measured by surrounding it with a region of interest (ROI).

### 3.8. In Vivo Therapeutic Experiment

When tumor volumes of the mice reached 100–150 mm^3^, the mice were randomly divided into five groups (five mice/group; total 25 mice): (i) no treatment (Control); (ii) 100 J/cm^2^ of NIR light irradiation without WL12-IR700 (NIR light); (iii) intravenous injection of WL12-IR700 (30 µg/mouse) without NIR light irradiation (Drug); and (iv) or (v) intravenous injection of WL12-IR700 (10 or 30 µg/mouse) and 100 J/cm^2^ of NIR light irradiation (PIT_10 µg or PIT_30 µg). After checking for the accumulation of WL12-IR700 using a Pearl Trilogy (LI-COR Biosciences, Lincoln, NE, USA), NIR light irradiation using an ML7710 laser system (Modulight; Tampere, Finland) and an FD1 frontal light distributor model (Medlight; Ecublens, Switzerland) in the PIT groups was performed 3 h after administration (laser parameters; spot diameter: 1.0 cm, irradiance: 150 mW/cm^2^, treatment time: 667 s). The tumors of the mice were measured three times per week, and mice with a tumor volume > 2000 mm^3^ were treated as dead.

### 3.9. Statistical Analysis

The obtained results are presented as means with standard deviations. Statistical analysis was performed using EZR software version 1.68 [39]. In the cell viability assay, the non-irradiated and NIR light irradiated groups were compared in vitro using Welch’s *t*-test. In the accumulation study, the average radiant efficiency was compared using the paired *t*-test adjusted by the Holm method after repeated-measures Analysis of Variance (ANOVA). In the therapeutic experiment, the tumor volumes of each group were compared using Dunnett’s multiple comparison test after a one-way ANOVA with respect to the control group. The overall survival rate of each group was compared with that of the control group using the log-rank test. Statistical significance was set at *p* < 0.05.

## 4. Conclusions

We developed a novel peptide-based NIR-PIT drug targeting PD-L1 using WL12, which binds to PD-L1. We synthesized a WL12-IR700 conjugate and evaluated it in cells and tumor-bearing mice. In in vitro studies, PIT-like morphological changes in cells and decreased cell viability were observed in an NIR light dose- and drug concentration-dependent manner. Additionally, in animal experiments, the PIT groups showed significant suppression of tumor growth and extension of overall survival rates compared with the control group. These results indicate the potential of NIR-PIT using WL12-IR700 targeting PD-L1.

## Data Availability

Data is contained within the article or Supplementary Materials.

## Supplementary Materials

The following supporting information can be downloaded at: https://www.mdpi.com/article/10.3390/molecules31020302/s1, Figure S1: Elution profiles on a RP-HPLC and MS data of WL12 (**1**) and WL12-IR700 (**2**); Figure S2: Absorption and fluorescence spectra of WL12-IR700 and IR700; Figure S3: Representative fluorescence images of WL12-IR700 and IR700 at 1, 3, and 6 h post-injection.

## Abbreviations

The following abbreviations are used in this manuscript:

NIR-PIT	Near-infrared photoimmunotherapy
IR700	IRDye700DX
EGFR	Epidermal growth factor receptor
PD-L1	Programmed death-ligand 1
PD-1	Programmed death-1
NSCLC	Non-small cell lung cancer
PET	Positron emission tomography
Fmoc	9-Fluorenylmethyloxycarbonyl
SPPS	Solid-phase peptide synthesis
NHS	*N*-hydroxysuccinimide
DIC	Differential interference contrast
PI	Propidium iodide
CCK-8	Cell Counting Kit-8
HPLC	High performance liquid chromatography
TFA	Trifluoroacetic acid
TEAA	Triethylammonium acetate
ESI	Electrospray ionization
MS	Mass spectra
DMF	*N*, *N*-dimethylformamide
EDT	1,2-Ethanedithiol
TIS	Triisopropylsilane
TEA	Triethylamine
RP	Reverse phase
DIPEA	*N*, *N*-diisopropylethylamine
DMSO	Dimethyl sulfoxide
ATCC	American Type Culture Collection
DMEM	Dulbecco’s modified Eagle’s medium
FBS	Fetal bovine serum
RPMI	Roswell Park Memorial Institute
PBS	Phosphate-buffered saline
ROI	Region of interest
ANOVA	Analysis of Variance
