# Supplementary material for: Development of a Peptide-Based Photoimmunotherapy Drug Targeting PD-L1
Source: Molecules. 2026 Jan 14;31(2):302. doi: 10.3390/molecules31020302 (PMC12843834; doi:10.3390/molecules31020302)

Supplementary Materials

Development of a peptide-based photoimmunotherapy drug targeting PD-L1

Takuya Otani, Naoya Kondo, Ayaka Kanai, Hirofumi Hanaoka*

Near InfraRed Photo-ImmunoTherapy Research Institute, Kansai Medical University, Hirakata, Osaka 573-1010, Japan

Table of Contents

Figure S1: Elution profiles on a RP-HPLC and MS data of WL12 (**1**) and WL12-IR700 (**2**) S2–S3

Figure S2: Absorption and fluorescence spectra of WL12-IR700 and IR700 S4

Figure S3: Representative in vivo fluorescence images of WL12-IR700 and IR700 S5

Figure S1: Elution profiles on a RP-HPLC and MS data of WL12 (**1**) and WL12-IR700 (**2**)

1

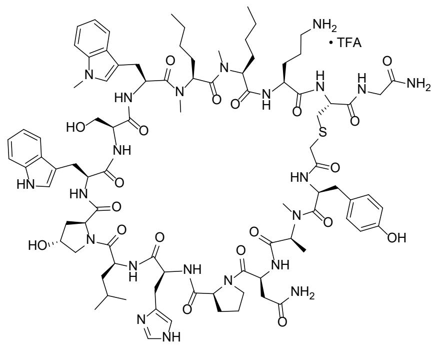

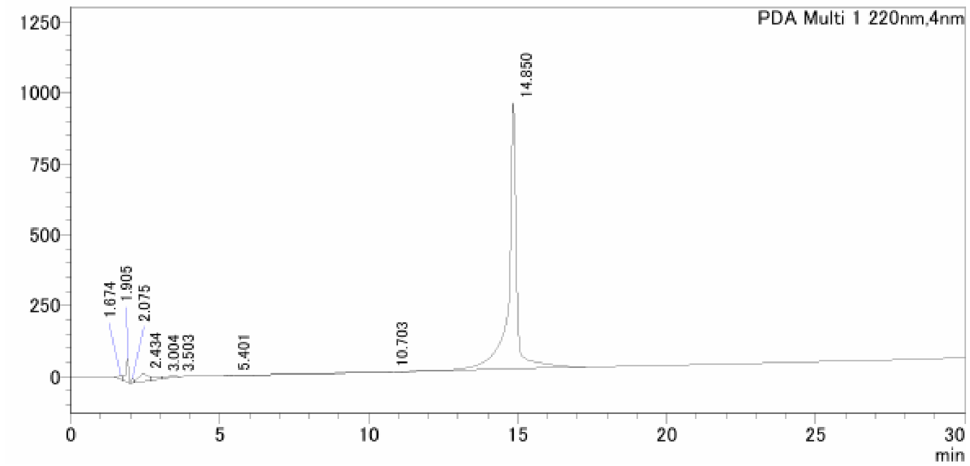

HPLC conditions: COSMOSIL Packed Column 5C18-AR-II (4.6 ID × 150 mm), detection at 220 nm, flow rate: 0.9 mL/min, run time: 30 min, solvent A: 0.1% TFA–H_2O; solvent B: 0.1% TFA–CH_3CN, linear gradient of 30–60% solvent B, $\boldsymbol{t}_{\mathbf{R}}$ 14.850 min.

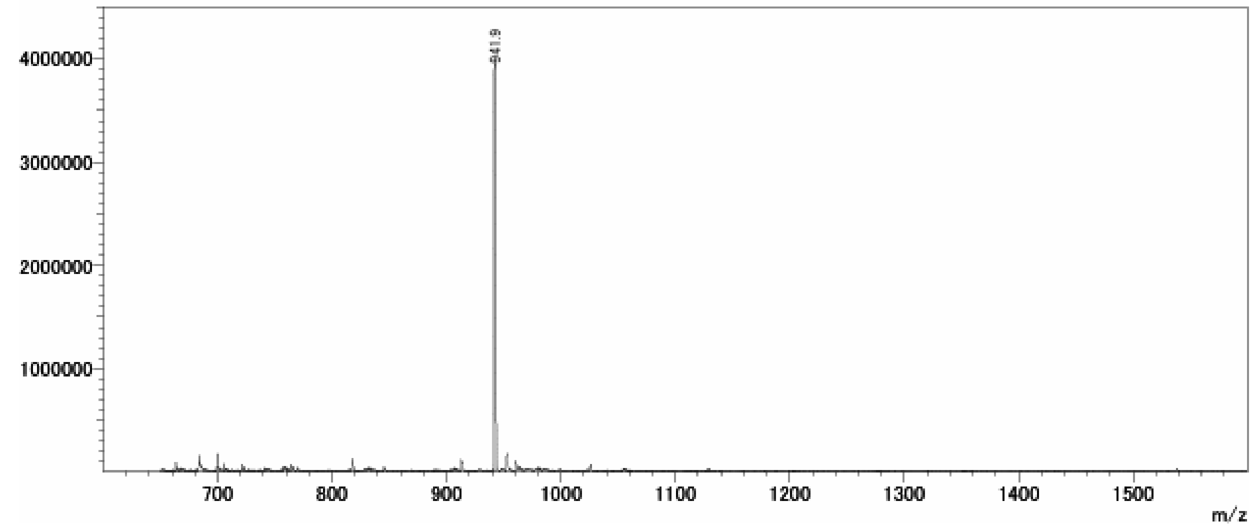

ESI-MS *m/z* calculated for $C_{91}H_{128}N_{22}O_{20}S$: 1880.9 (TFA desalted), recorded a peak at $[M+2H]^{2+}$ *m/z*: 941.9.

2

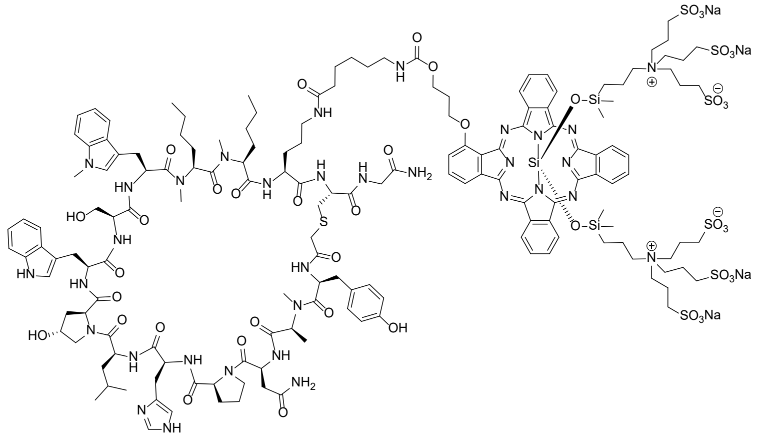

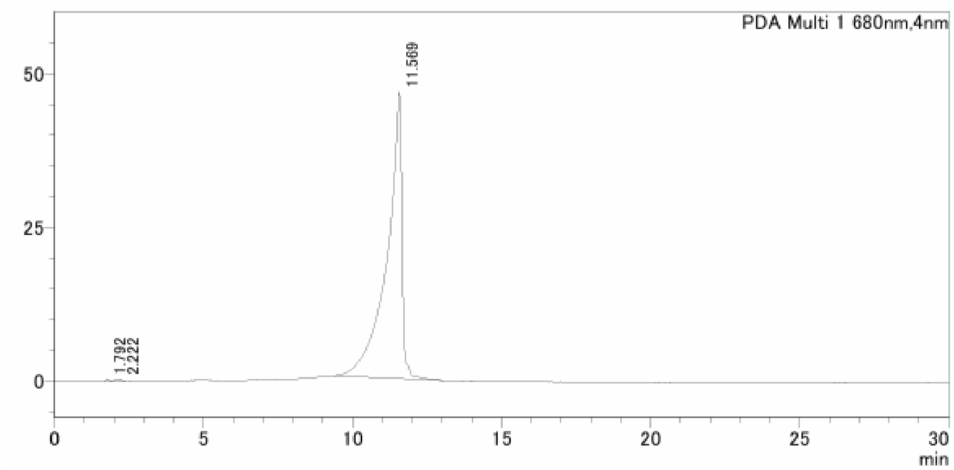

HPLC conditions: COSMOSIL Packed Column 5C18-AR-II (4.6 ID × 150 mm), detection at 680 nm, flow rate: 0.9 mL/min, run time: 30 min, solvent A: 0.1M TEAA–H_2O; solvent B: CH_3CN, linear gradient of 35–65% solvent B, $\boldsymbol{t}_{\mathbf{R}}$ 11.569 min.

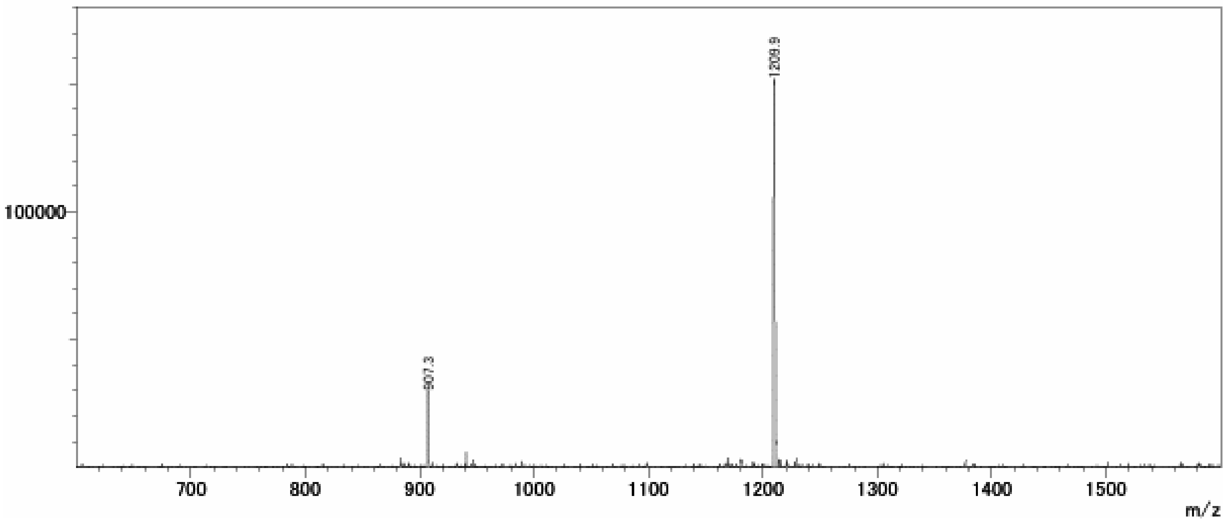

ESI-MS *m/z* calculated for $C_{161}H_{219}N_{33}O_{44}S_7Si_3{}^{4-}$: 3626.3 ($Na^+$ desalted), recorded a peak at $[M]^{4-}$ *m/z*: 907.3, $[M+H]^{3-}$ *m/z*: 1209.9.

Figure S2: Absorption (blue) and fluorescence (red) spectra of WL12-IR700 and IR700.

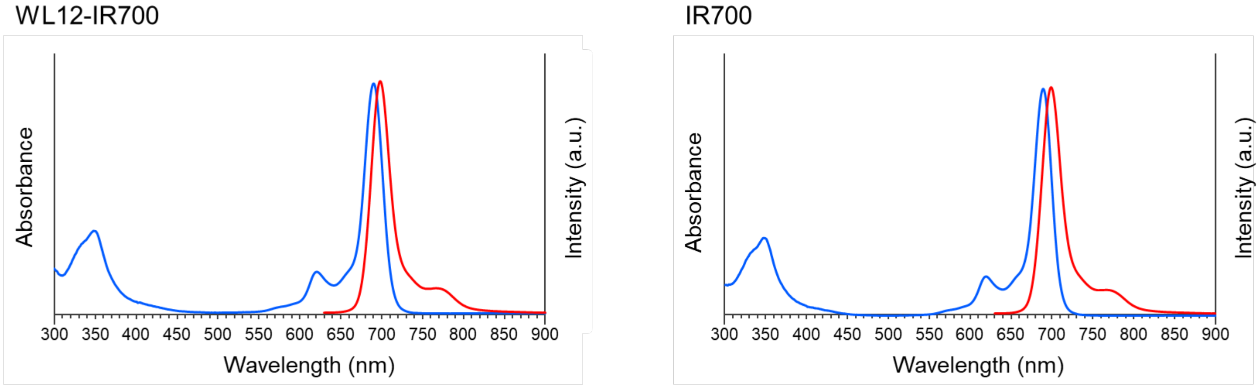

Conditions of absorption spectra; concentration: 1.0 μM, range: 900–300 nm, measurement data interval: 1 nm, λ_{max} = 690 nm (WL12-IR700) and 689 nm (IR700).

Conditions of fluorescence spectra; concentration: 0.5 μM, excitation wavelength: 610 nm, range: 630–900 nm, measurement data interval: 1 nm, λ_{max} = 698 nm (WL12-IR700) and 699 nm (IR700).

Figure S3: Representative in vivo fluorescence images of WL12-IR700 and IR700 at 1, 3, and 6 h post-injection. These are overlays of bright field image and fluorescence image. Tumors and its contralateral side were surrounded by ROI under bright field image, and the average of radiant efficiency per area was calculated. In addition, the ratio between the tumor and its contralateral side was calculated (lower graph).

<u>WL12-IR700</u>

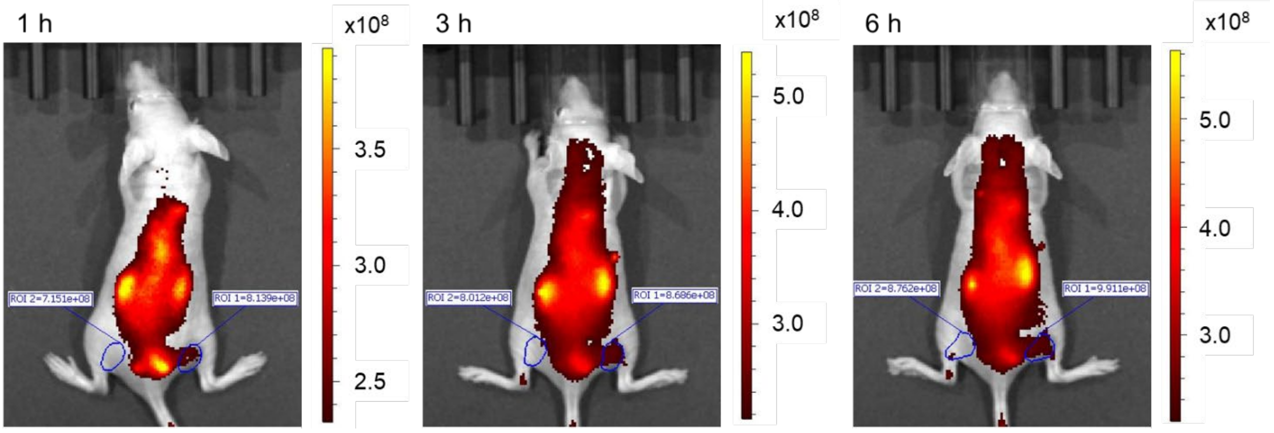

<u>IR700</u>

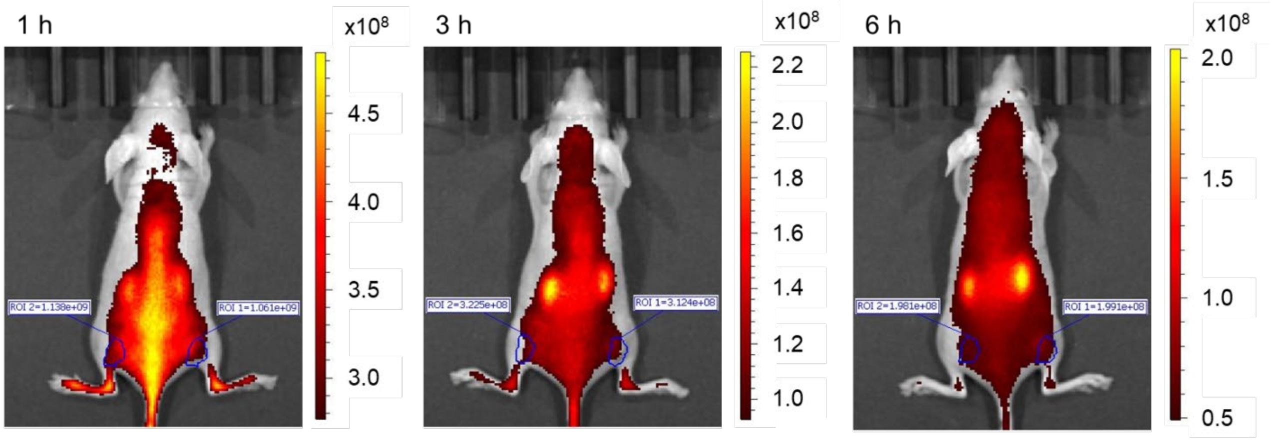

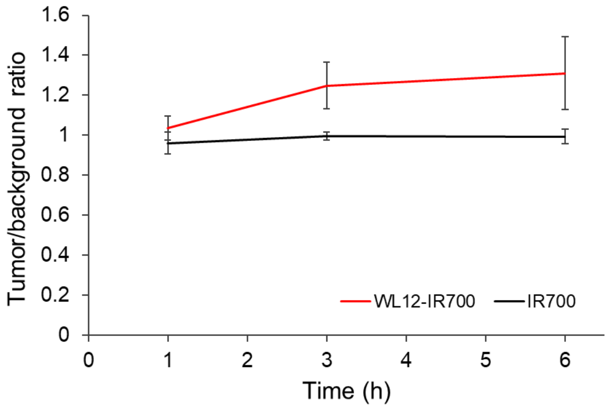

Supplement: Supplementary file 1 [file molecules-31-00302-s001.zip › molecules-3979933-supplementary.pdf]
